# Bioactive Mimetics of Conotoxins and other Venom Peptides

**DOI:** 10.3390/toxins7104175

**Published:** 2015-10-16

**Authors:** Peter J. Duggan, Kellie L. Tuck

**Affiliations:** 1 CSIRO Manufacturing, Bag 10, Clayton South, VIC 3169, Australia; 2 School of Chemical and Physical Sciences, Flinders University, Adelaide, SA 5042, Australia; 3 School of Chemistry, Monash University, Clayton, VIC 3800, Australia

**Keywords:** venom peptides, toxins, conotoxins, peptidomimetics, N-type calcium channel, Ca_v_2.2

## Abstract

Ziconotide (Prialt^®^), a synthetic version of the peptide ω-conotoxin MVIIA found in the venom of a fish-hunting marine cone snail *Conus magnus*, is one of very few drugs effective in the treatment of intractable chronic pain. However, its intrathecal mode of delivery and narrow therapeutic window cause complications for patients. This review will summarize progress in the development of small molecule, non-peptidic mimics of Conotoxins and a small number of other venom peptides. This will include a description of how some of the initially designed mimics have been modified to improve their drug-like properties.

## 1. Introduction

A wide range of species from the animal kingdom produce venom for use in capturing prey or for self-defense. Animal venoms typically consist of a complex mixture of proteins, peptides and small molecules [1]. Venom peptides in particular have been intensively studied following the discovery of fascinating pharmacological properties of peptides isolated from marine mollusks and terrestrial snakes and arthropods. The sheer number of bioactive venom peptides produced by species from the animal kingdom is astronomical [1] and these represent a vast and largely untapped source of new research tools and therapeutic leads. Peptides found in the venom of the fish-hunting cone snails of the genus *Conus* have been the focus of considerable attention, largely due to their often powerful and selective modulating effects on human ion channels. While a synthetic version of one of these *Conus* peptides (ω-conotoxin MVIIA, Ziconotide (Prialt^®^)) is now in limited clinical use, the biophysical properties of peptides in general makes them unsuitable for use as drugs. *Conus* peptides have, however, served as the inspiration for a number of bioactive discovery programs where the objective is to develop drug-like small molecules that mimic the biological activity of the natural peptide. Presented in this review is a discussion of the approaches used by researchers in this area and progress to date. Compared to conotoxin mimics, the mimicry of venom peptides from other sources has been more limited. Publically disclosed efforts with snake peptides and a peptide from the sun anemone will be described. The most successful of all of these programs has been the development of Tirofiban (Aggrastat^®^), an anti-platelet drug used to reduce the rate of thrombotic cardiovascular events and inspired by a tripeptide fragment of an anti-coagulant found in the venom of the African saw-scaled viper *Echis carinatus.*

## 2. Peptidomimetics

Peptide mimics or peptidomimetics are compounds that possess similar biological activity and/or 3D topography as a native peptide or protein fragment. Peptidomimetics typically interact with a biological target in a similar manner to the native peptide and as a result they exert a similar biological effect [2]. Peptidomimetics are commonly designed to overcome the bioavailability and instability issues associated with peptides and proteins. Three distinct types of peptidomimetics have been classified [3,4,5]. Type-I peptidomimetics, also known as structural mimetics, are peptide backbone mimetics, where the local topography is analogous to the native peptide and functionalities required to interact with the enzyme or receptor are present. Type-II peptidomimetics, also known as functional mimetics, interact with the enzyme or receptor of interest in a related way to the active peptide but are not necessarily structurally related to the peptide. Type-III peptidomimetics, also known as functional-structural mimetics, have a core scaffold structure that is different from the active peptide, and from which projects amino acid side chain mimics in a well-defined spatial orientation, simulating the way the native peptide interacts with the biological target.

One approach to the development of type-III mimetics involves the mimicry of the α,β-bond vectors of the amino acid residues responsible for a peptide’s biological activity. This method was promulgated by Bartlett and Lauri [6] and is the fundamental tenet of the Computer-Assisted Vector Evaluation and Target Design (CAVEAT) program [7]. Much of the biological activity elicited by an active peptide results from the interaction of the side chain *termini* with the receptor, and when detailed structural information about this interaction is absent, it is very difficult to predict the exact spatial orientation of these termini when bound. The position and direction of the first side chain bonds that project from the peptide backbone, the α,β-bond vectors, are however, usually much better defined. If a reliable solution state structure or computational model of the peptide is available, the α,β-bond vector approach can be used to design type-III mimetics. At their core, these compounds have a semi-rigid molecular scaffold from which bonds that are positioned to simulate the location and direction of the α,β-bond vectors of the key amino acids side chains on the active peptide are projected. Amino acid side mimics are attached to α,β-bond replicas with the expectation that, as with the active peptide, the termini of these functionalities will find the desired binding site within the receptor.

## 3. Pain Blocking Conotoxins and Their Mimetics

### 3.1. Conotoxins

Conotoxins are neurotoxic peptides isolated from the venom of marine cone snails from the genus *Conus*. To date over 700 species from this genus have been described, with the venom of each containing a unique and diverse mixture of pharmacologically active compounds [8,9,10,11]. Conotoxins are small, structurally defined peptides that range in size from eight to thirty amino acid residues and typically have one or more disulfide bonds. The fish-hunting cone snails have the most poisonous venom and there have been over 30 deaths as a result of cone snails envenomation [10]. The majority of these deaths are due to the cone snail *Conus geographus*, whose sting can cause paralysis within 30 min and coma or death within five hours [12]. Interestingly, in these unfortunate cases the victim has been reported to die a “painless death” [10]. The potency and analgesic activity of the *Conus* venoms has motivated a number of research groups to isolate and identify the pharmacologically active components in the venom, with Olivera and coworkers being pioneers in this field. There are a number of excellent reviews that summarize this research [13,14,15,16,17]. The comprehensive pharmacology of conotoxin peptides [9,10,16,17,18,19,20,21,22] has also been comprehensively reviewed, and hence will not be described here in detail.

The nomenclature for conotoxins was first proposed in 1985 [23] and further refined in 1988 [15]: When the conotoxin has a known mode of action, then a Greek letter prefix is used to designate its pharmacological action. This is then followed by “-conotoxin”, a one or two letter code to assign the *Conus* species, a Roman numeral to indicate the Cys arrangement and finally an upper case letter that allows conotoxins of the same class, isolated from the same species, to be differentiated [13,15,23]. For example, ω-conotoxin GVIA targets voltage gated calcium channels (ω), is a peptide from the *Conus* species *Conus geographus* (G) and has a class VI Cys pattern (C–C–CC–C–C). Conotoxins of particular relevance to this article are the ω-conotoxins and μ-conotoxins. More recently conotoxins have been classed according to their superfamily. Peptides within a superfamily have a highly conserved amino acid sequence and share the same disulfide connectivity [24,25]. The ω-conotoxins belong to the O1-superfamily of which κ-, δ-, and µO-conotoxins are also members, and the μ-conotoxins are members of the M-superfamily. For a recent review on conotoxin gene superfamilies, see Robinson and Norton [26].

**Figure 1:**
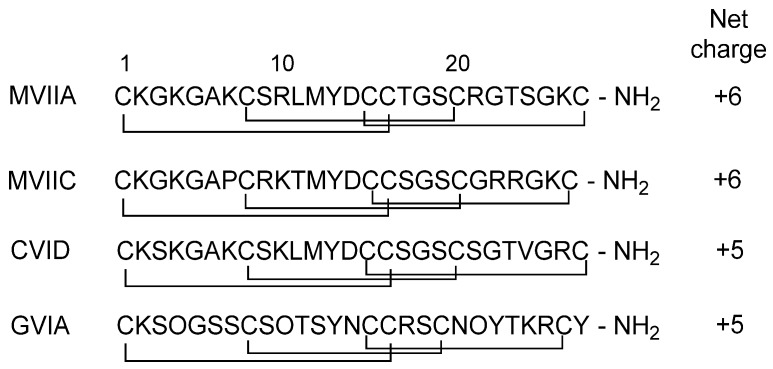
Single letter amino acid sequences of ω-conotoxin MVIIA, MVIIC, CVID and GVIA; disulfide bridges are indicated by solid lines, O = hydroxyproline.

**Figure 2:**
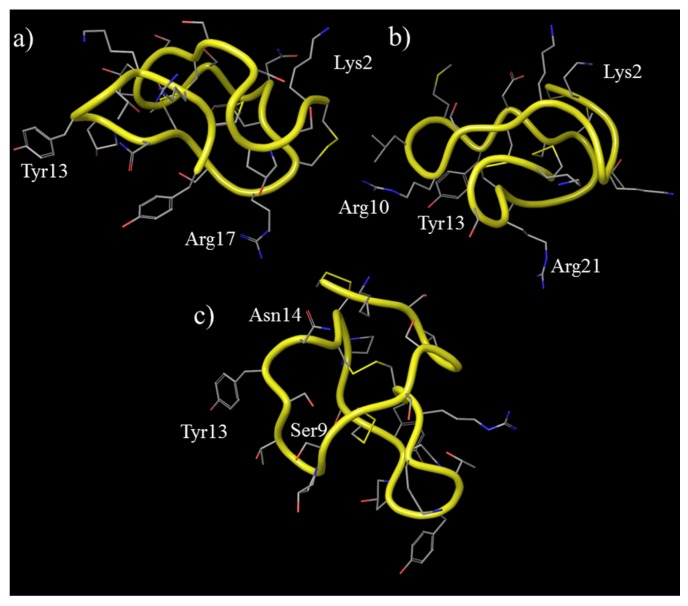
Three-dimensional structures of ω-conotoxins that are relevant to this review. The peptide backbone structure is shown as a yellow tube, the side chain residues are shown as thin tubes and are colored according to the atom type, and hydrogen atoms are not shown. The amino acid side chains thought to be important for biological activity are highlighted. (**a**) ω-conotoxin GVIA (PDB code: 2cco); (**b**) ω-conotoxin MVIIA (PDB code: 1ttk); and (**c**) ω-conotoxin CVID (coordinates obtained from reference [29]).

ω-Conotoxins contain six Cys residues and three disulfide bridges. To date 21 ω-conotoxins peptides have been identified with net charges ranging from +5 to +7 [21,27,28]. The most well studied ω-conotoxins are the N-type calcium channel (Ca_v_2.2) blockers MVIIA and GVIA, and the P/Q-type calcium channel (Ca_v_2.1) blocker CVID. The amino acid sequences of these peptides are shown in Figure 1 as well as the sequence of MVIIC, a Ca_v_2.1 blocker. The backbone structure of ω-conotoxins MVIIA, GVIA and CVID, determined from Nuclear Magnetic Resonance (NMR) spectroscopy experiments, are shown in Figure 2.

Voltage-gated calcium ion channels (VGCC) control a number of physiological functions including chronic and neuropathic pain. For recent reviews on the topic see references [30,31,32,33,34,35,36,37]. VGCC can be broadly divided into two classes; the low-voltage activated and high-voltage activated channels. The N-type (Ca_v_2.2) and P/Q-type calcium channels (Ca_v_2.1) are members of the high-voltage activated channels with the ω-conotoxins selectively targeting these channels. The ability of conotoxins such as MVIIA, GVIA and CVID to selectively block Ca_v_2.2 channels is of significant interest as these channels are implicated in refractory pain states such as neuropathic pain. By contrast, Ca_v_2.1 channels occur at the neuromuscular junction and the inhibition of these channels can produce a number of nasty pathological effects. ω-Conotoxin MVIIC is known to inhibit Ca_v_2.1 channels.

Μ-Conotoxins contain six Cys residues, three disulfide bridges, and have a class III Cys pattern (CC–C–C–CC). To date, 20 μ-conotoxins peptides have been identified [38]. μ-Conotoxins selectively block voltage-gated sodium channels (VGSCs).

### 3.2. Commonly Used Assays

Before discussing the development of conotoxin mimetics as potential pain blockers, a summary of typical bioassays used to assess these mimetics is presented here.

#### 3.2.1. Rat Vas Deferens

It has been shown that the twitch contractions of the rat vas deferens, in response to electrical field simulation, are controlled by release of neurotransmitters governed by the Ca_v_2.2 channel [14,18,39]. These electrically induced muscle contractions are inhibited by ω-conotoxin GVIA and it is proposed that this is due to the peptide binding to the presynaptic sites on the Ca_v_2.2 channels [14,18,39]. Consequently, this assay was used in early studies to assess the structure–function of ω-conotoxin GVIA [40,41] and CVID [42], and the potency of peptidomimetics of ω-conotoxin GVIA [43].

#### 3.2.2. Radioligand Displacement Assay

The radioligand binding assay was first used by Snyder and coworkers in 1988 to characterize the binding of ω-conotoxin GVIA in the brain, using membranes from rat brains and ^125^I-labeled ω-conotoxin GVIA [44]. This technique has been extended to the development of a radioligand displacement assay, which can be used to gauge the ability of compounds to bind to rat brain Ca_v_2.2 channels [45]. After the preparation of crude rat brain membrane, the labeled peptide and a range of ligand concentrations are added [45], and a dose-response is then determined by counting the residual ^125^I-labeled ω-conotoxin GVIA that remains associated with the membrane. The binding affinity of small molecule mimetics of ω-conotoxin GVIA to the Ca_v_2.2 channel has been determined by this method [46,47,48,49,50,51]. It is assumed that a compound that is able to displace ^125^I-labeled ω-conotoxin GVIA from rat brain membrane does so by binding to the same part of the Ca_v_2.2 ion channel as GVIA and hence should have the same pain-blocking effect. This may not always be true as other factors such as allosteric effects could cause the labeled GVIA to be released from the brain membrane. Nonetheless, this assay has served as a useful initial screen for Ca_v_2.2 channel affinity and has led to the discovery of compounds with functional activity in, for example, electrophysiological assays.

#### 3.2.3. FLIPR assay with SH-SY5Y Neuroblastoma Cells

In recent years a functional FLIPR (Fluorescence Imaging Plate Reader) assay has replaced the radioligand displacement assay as the high-throughput assay of choice for the screening of Ca_v_2.2 blockers. Neuroblastoma cells are a good cellular model for monitoring VGCC [52]. The magnitude of intracellular calcium flux elicited by the addition of KCl and CaCl_2_ is determined by measuring the change in fluorescence of a calcium-sensitive dye and can be used to gauge the level of inhibition of calcium channels by added compounds. For Ca_v_2.2 inhibition studies, SH-SY5Y neuroblastoma cells that endogenously express human Ca_v_2.2 channels in a physiologically relevant context, are briefly incubated with varying concentrations of the compounds of interest, in the presence of saturating concentrations of the L-type calcium channel blocker nifedipine. The Ca^2+^ responses produced by KCl-depolarization are measured and dose-response curves enable calculation of IC_50_ values [51,53,54]. The major drawbacks of the radioligand displacement assay—The requirement for radioisotopes and the fact that it is not a functional assay—are negated with the FLIPR assay. Furthermore, the FLIPR assay results are reported to be a better predictor of activity in electrophysiological assays (for information on electrophysiological methods see below). Estimated IC_50_ values for the functional inhibition of calcium channels obtained from the FLIPR assay and electrophysiology experiments have, in some cases, been observed to be in close agreement, whereas the IC_50_ values obtained from the radioligand displacement assay can over estimate functional activity by up to an order of magnitude [51].

#### 3.2.4. IMR-32 Human Neuroblastoma Cell Assay

IMR-32 human neuroblastoma cells were first used to investigate the binding of ω-conotoxins to VGCC in 1988 (see reference [55] and later references [52,56]). The IMR-32 human neuroblastoma cell assay has some similarities to the SH-SY5Y neuroblastoma cell assay described above, both are fluorescent based assays, intracellular calcium flux elicited by the addition of K^+^, and both assays include the L-type calcium channel blocker nifedipine. To date, there is only one report of this assay being used to determine the binding affinity of small molecule mimics of an ω-conotoxin and in this assay Indo-1 is used as the indicator [57].

#### 3.2.5. Electrophysiological Methods—Patch Clamp Technique

Electrophysiological methods have long been considered the “gold-standard” technique for studying ion channels. In the patch clamp technique, whole cell electrophysiological recordings are made using a glass micropipette electrode that is “clamped” to the surface of a cell expressing the ion channel of interest. For voltage gated ion channels like Ca_v_2.2, the channel can be cycled from the resting to the activated state by varying the electrical potential from a holding potential of ~−80 mV to more depolarized test potentials, and the current generated under the voltage clamp measured. The inhibition of such channel currents by potential channel blocking compounds can be observed by adding the compounds to the extra-cellular solution. Dose responses can be used to assess the relative effectiveness of channel blockers. Since the patch clamp technique can be used to cycle voltage-gated ion channels through resting, depolarized and inactivated forms, a compound’s effect on each of these states can be measured. This information can be used to optimize compounds for particular channel states relevant to certain disease pathologies like, for example, neuropathic pain.

### 3.3. ω-Conotoxin Mimetics

#### 3.3.1. ω-Conotoxin MVIIA

**Figure 3:**
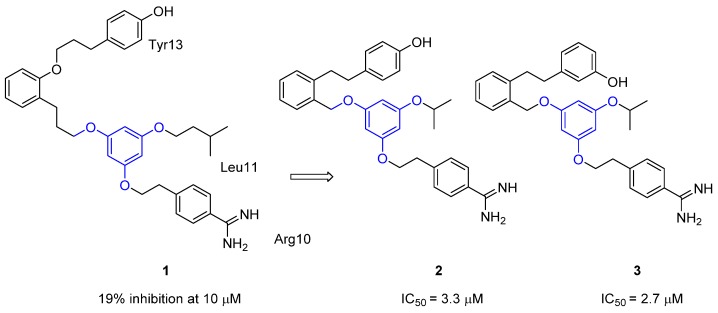
Non-peptide mimetics containing a dendritic scaffold. The dendritic scaffold is highlighted in blue. IC_50_ values are for Ca_v_2.2 activity in the IMR-32 assay [57,58].

The first report of a low molecular weight type-III peptidomimic of ω-conotoxin MVIIA was by the group of Horwell from Parke-Davis (now Pfizer) [58]. The ^1^H NMR solution structure of the peptide revealed the backbone structure of the peptide [59] and it was known that the residues required for binding were Lys2, Arg10, Leu11, Tyr13 and Arg21 (Neurex Corporation) [60]. Utilizing this information, Horwell *et al.* designed a dendritic scaffold that would enable the mimicry of three key amino acid residues [Arg10, Leu11 and Tyr13]. The first generation compound, the synthesized alkylphenyl ether (compound **1** in Figure 3) based on a phloroglucinol core [58], gave poor inhibition (19% at 10 μM) in an N-type IMR-32 human neuroblastoma cell assay [57]. The second generation compounds (**2** and **3**), whose design was also supported by molecular modeling, gave improved activity (IC_50_ values of 3.3 and 2.7 μM) [57]. This report was one of the first to use *in silico* design to assist with the construction of active compounds, thereby enabling the pharmacophore of the native peptide to be better replicated. The flexibility of the projected residues in these mimics was integral to their design as it was expected that their fluxional nature would allow them to “sample” a wider region of conformational space and hence would be more likely to able to adopt an optimal ion channel binding conformation.

**Figure 4:**
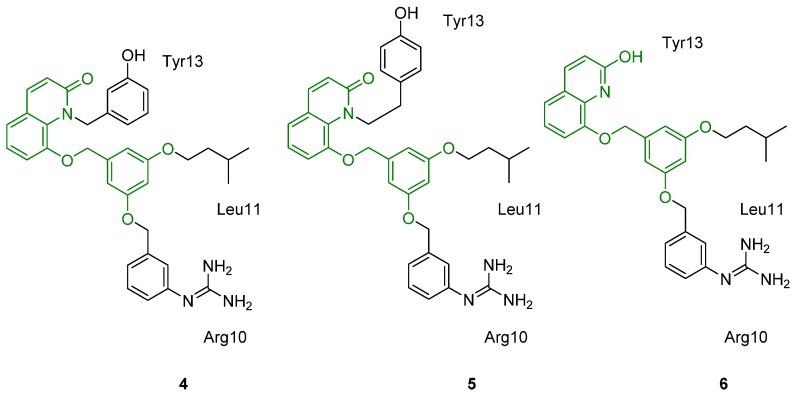
Non-peptide mimetic of ω-conotoxin MVIIA, using the scaffold core of 8-hydroxy-2-(1H)-quinolinone and 5-hydroxymethyl resorcinol. The scaffold core is highlighted in green [61].

In a follow-up report, three dendroid molecules were synthesized using the core of 8-hydroxy-2-(1H)-quinolinone attached to a 5-hydroxymethyl resorcinol (**4**–**6**, Figure 4) [61]. While these compounds were designed to be mimics of ω-conotoxin MVIIA, their ability to inhibit Ca_v_2.2 channels has not been reported.

#### 3.3.2. ω-Conotoxin CVID

**Figure 5:**
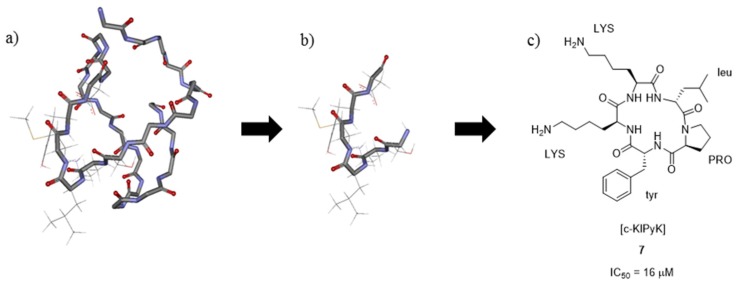
(**a**) Diagram of the three-dimensional structure of ω-conotoxin CVID with sidechains of residues **9**–**14** shown, coordinates obtained from reference [29]. (**b**) Backbone and sidechains of residue **9**–**14** alone; (**c**) Structure of [c-KlPyK] **7**, the most potent analogue discovered (L-amino acids are shown as uppercase letters and D-amino acids are represented by lowercase letters). The IC_50_ value was determined using the radioligand displacement assay with ^125^I-GVIA [64].

It has been shown that Leconotide, a synthetic version of CVID (also known as AM336 and CNSB004), is the most selective ω-conotoxin for the N-type (Ca_v_2.2) over the P/Q-type (Ca_v_2.1) calcium channel with 700,000-fold selectivity observed [42]. Furthermore, the native peptide has been shown to have a wider therapeutic window than Ziconotide (Prialt^®^) and has fewer cardiovascular side effects [62,63]. Lewis and coworkers developed small molecule cyclic pentapeptides as type-I mimics of ω-conotoxin CVID [64]. The ^1^H NMR solution structure of ω-conotoxin CVID showed that this conotoxin has two hydrogen bonds between loop 2 and 4 that stabilize loop 2 and provide extra rigidity [42]. Lewis and coworkers determined the C_α_–C_β_ vectors of the residues believed to be required for binding to the Ca_v_2.2 channel, concentrating on residues **9**–**14** that are found in loop 2 [64]. Simplification of this pharmacophore to the C_α_–C_β_ of residues **9**–**14** enabled virtual screening of a library of compounds and identification of the cyclic pentapeptide framework containing one or more D-amino acids (Figure 5). In total, twenty cyclic pentapeptides of this first series were synthesized and tested for their binding affinity to the Ca_v_2.2 channel using the radioligand displacement assay with ^125^I-GVIA. Two analogues [c-AKlMy] and [c-aKlMY] (L-amino acids are shown as uppercase letters and D-amino acids are represented by lowercase letters), gave IC_50_ values of 40 and 60 μM respectively. Based on these results a second series was designed and tested. This series, [c-KlPyK], [c-lKPyK] and [c-lPKyK], had improved rigidity due to the introduction of a proline residue and also possessed a second positive charge through the inclusion of a second lysine residue. The IC_50_ values for the second series of peptides ranged from 16 to 480 μM. The most potent analogue was [c-KlPyK] (compound **7** in Figure 5, IC_50_ = 16 μM). These compounds were also tested for their selectivity for N-type (Ca_v_2.2) over the P/Q-type (Ca_v_2.1) voltage gated calcium channel by testing their binding affinity in the radioligand displacement assay with ^125^I-MVIIC. ω-Conotoxin MVIIC is known to inhibit Ca_v_2.1. None of the peptides were found to displace ^125^I-MVIIC at concentrations below 300 μM and it was thus concluded that these cyclic peptides were selective for N-type voltage gated calcium channels.

As the cyclic pentapeptides discussed above mimic the binding of ω-conotoxin CVID and are selective against for N-type calcium channels, there was great hope that small molecules could be designed and developed to mimic ω-conotoxin CVID. However, since this original report by Lewis and coworkers in 2004 [64] there have been no further reports of CVID peptidomimetics.

#### 3.3.3. ω-Conotoxin GVIA

**Figure 6:**
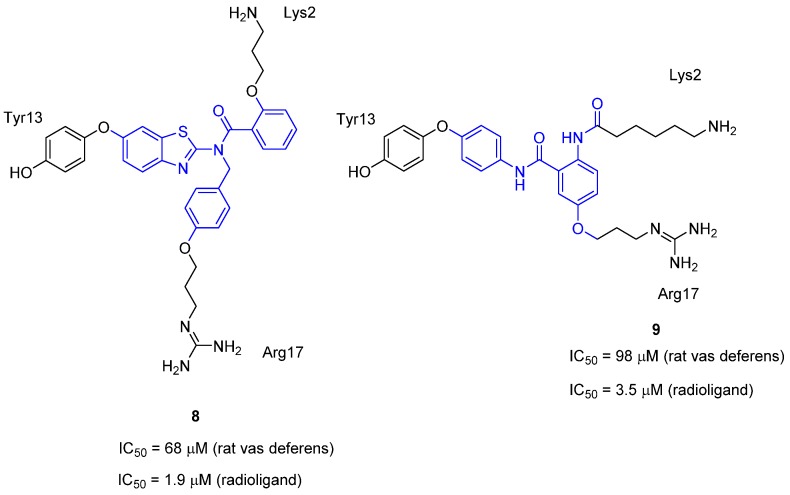
Initial type-III peptidomimetics of ω-conotoxin GVIA. Ca_v_2.2 activity was determined using the rat vas deferens assay [43] and later using the radioligand displacement assay with ^125^I-GVIA [46,47]. The core scaffold is colored blue.

**Figure 7:**
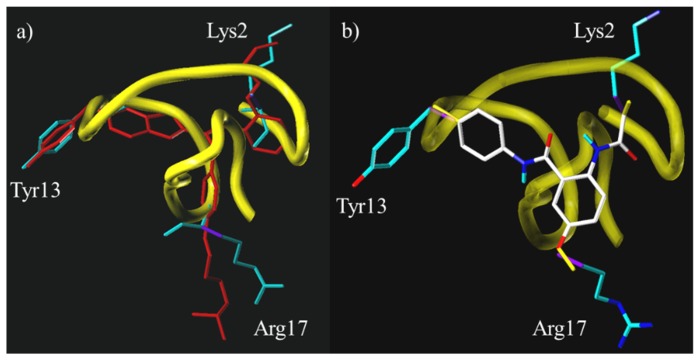
Backbone structure of ω-conotoxin GVIA (yellow) with the amino acid side chains Lys2, Tyr13, Arg17 shown in teal; (**a**) overlaid with a model of the benzothiazole mimetic **8** [28]—Reproduced with permission; and (**b**) overlaid with X-ray crystal structure of the anthranilamide scaffold core without side chain mimics. The α,β-bond vectors are shown in purple and the bonds mimicking the α,β-bond vectors are shown in yellow [47]—Reproduced with permission.

Of all the ω-conotoxins, ω-conotoxin GVIA has been the subject of the most reports of type-III mimetics, firstly by the group of Baell and more recently from the group of Duggan and Tuck. The initial report of a type-III GVIA peptidomimetic was by Baell *et al.* in 2001 [43]. Three non-planar, rigid scaffolds were designed using the data from the NMR solution structure of ω-conotoxin GVIA, utilizing the C_α_–C_β_ bond vector approach combined with interactive *de novo* design. It was known that the residues Lys2 and Tyr13 were crucial for potency and to a lesser extent the residues Arg17, Tyr22 and Lys24 [40]. With this in mind, Baell *et al.* concluded that a three-point mimic must include Lys2 and Tyr13, but it was unclear which of the remaining three residues should be included. Guided by molecular modeling and scaffold design, it was decided that the third residue would be Arg17. Mimetics based on two out of the three proposed scaffolds, the benzothiazole derivative **8** and the anthranilamide derivative **9**, were synthesized and were found to have activity in the rat vas deferens assay, with IC_50_ values of 68 μM and 98 μM, respectively, being obtained (see Figure 6 for relevant compound structures). Subsequent testing of these compounds for Ca_v_2.2 activity using the radioligand displacement assay with ^125^I-GVIA gave the IC_50_ values of 1.9 μM [46] and 3.5 μM [47], respectively. An overlay of the benzothiazole and anthranilamide core scaffolds respectively with the NMR solution structure of ω-conotoxin GVIA is shown in Figure 7. This figure demonstrates that the benzothiazole mimetics and the anthranilamide mimics possess the correct geometry to ensure that the side chain mimics are positioned to replicate the location and direction of the α,β-bond vectors of the key amino acids side chains.

**Figure 8:**
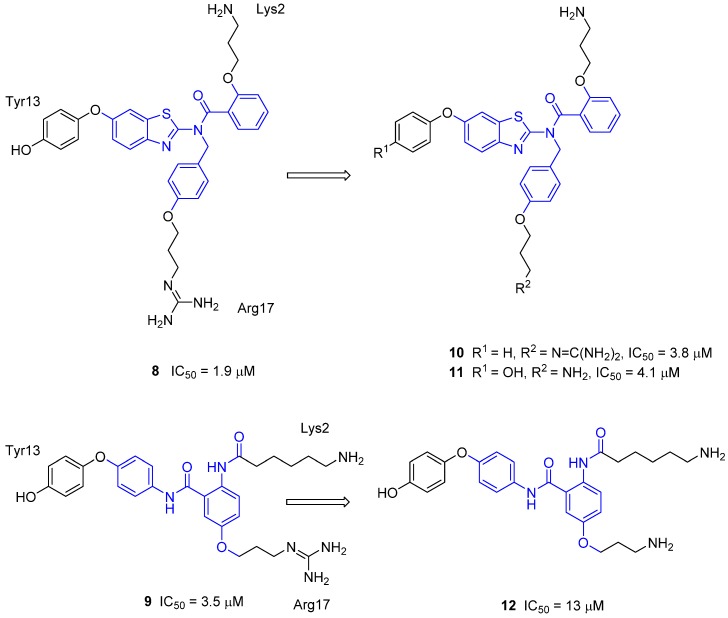
Exploration of the benzothiazole and anthranilamide scaffolds, Ca_v_2.2 activity was determined using the radioligand displacement assay with ^125^I-GVIA [46,47]. The scaffold core is colored blue.

Encouraged by the activity of benzothiazole derivative **8** and the anthranilamide derivative **9**, Baell, Duggan and coworkers synthesized analogues of both in order to start to build up structure-activity relationships [46,47]. Three benzothiazole analogues were tested for their binding affinity to the Ca_v_2.2 channel using the radioligand displacement assay with ^125^I-GVIA. The structures of two analogues are shown in Figure 8 [46]. Conversion of the original Tyr side chain to a Phe mimic, by removal of the phenolic OH group, gave compound **10**, which resulted in a negligible loss of activity (Figure 8). Limited activity loss was also observed when the guanidinium moiety was truncated to a primary amine (compound **11**). Interestingly, these benzothiazole analogues (**8**, **10** and **11**) showed a 20–25-fold selectivity for the N-type (Ca_v_2.2) over the P/Q-type (Ca_v_2.1) voltage gated calcium channel. In the anthranilamide series, truncation of the guanidinium to the primary amine was also investigated (Figure 8) [47]. Compound **9** was 30-fold more selective for Ca_v_2.2 over Ca_v_2.1. A negligible loss of activity was observed for **12** and this analogue was observed to be 13-fold selective for Ca_v_2.2 over Ca_v_2.1.

**Figure 9:**
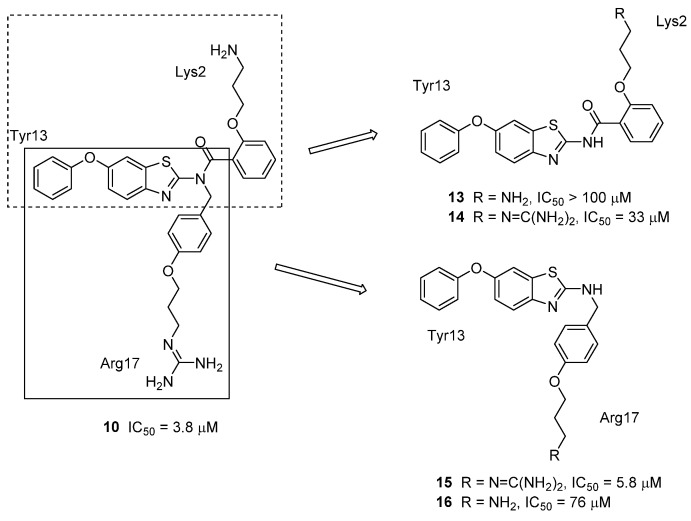
Truncation of the original benzothiazole scaffold **10** resulting in two-point mimetics **13**–**16**. Ca_v_2.2 activity was determined using the radioligand displacement assay with ^125^I-GVIA [50].

Further investigation of the benzothiazole scaffold by Duggan and Tuck resulted in the synthesis of the truncated analogues **13**–**16** [50]. Previous work had shown that rotation around the *N*-benzyl bond resulted in two main conformations; the Arg17 side chain mimic could point up or down with respect to the benzothiazole ring and hence this side chain had the potential to mimic the Lys2 side chain of GVIA. Truncation of compound **10** resulted in analogues that have a significantly lower molecular weight, and could be synthesized in 6–7 fewer steps (Figure 9). However, the truncated Tyr13-Lys2 mimic **13** was found to be inactive. The corresponding guanidylated compound **14** showed improved activity, however this was still ten-fold lower than that of the three-point mimic **10**. Conversely, the truncated Tyr13-Arg17 mimic **15** showed comparable activity to the parent analogue **10**, though taking the guanidinium functional group back to a primary amine (compound **16**) resulted in a significant loss of activity. This finding confirmed the important contribution that the guanidinium group makes to the observed activity of these benzothiazole compounds and showed that the truncated mimic, a two-point mimic, could have comparable activity to a more complex, higher molecular weight compound.

**Figure 10:**
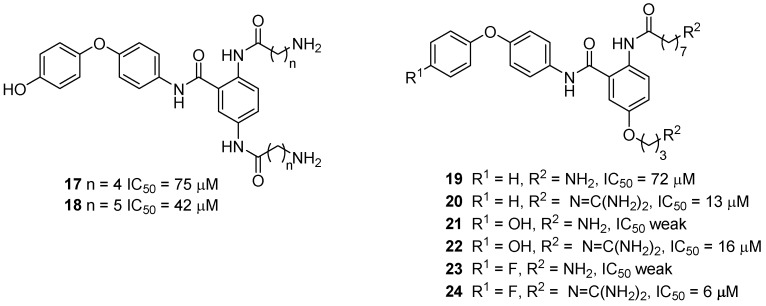
Exploration of the anthranilamide scaffold, only selected compounds are shown. Ca_v_2.2 activity was determined using the radioligand displacement assay with ^125^I-GVIA [48,49].

Additional work by Duggan and Tuck led to the synthesis of two amino-anthranilamide analogues that incorporate an amide linkage in the Arg17 mimic rather than the ether linkage (compounds **17** and **18**, Figure 10). However, while Ca_v_2.2 activity was retained, the potency was relatively weak and as such no further analogues of this type were investigated [48]. It was noted that long-chain aliphatic amines like dodecylamine had strong calcium channel affinity [65], and in light of this Duggan and Tuck undertook a comprehensive study into the structure activity relationship (SAR) relevant to the alkyl chain length of the anthranilamide derivative **12**. By varying the alkyl length of the ether and amide it was discovered that the optimum linker length was *n* = 3 and *n* = 7, respectively, although other linker lengths were well tolerated. They also explored removal of the *p*-hydroxyl substituent in **12** and replacement of the *p*-hydroxyl substituent with a *p*-fluoro substituent [49]. Fluorine, a hydroxyl surrogate, is advantageous as phenoxy groups are very quickly metabolized in the liver and replacement of the phenoxy group with fluorine prevents this metabolism. This study also investigated if a single guanidinium group was required for good activity or if diguanidinylated compounds were appropriate. It was found that the diguanidinylated compounds were the most active, deletion of the hydroxyl substituent had little influence on activity and the analogue with the *p*-fluoro substituent was the most active compound prepared.

**Figure 11:**
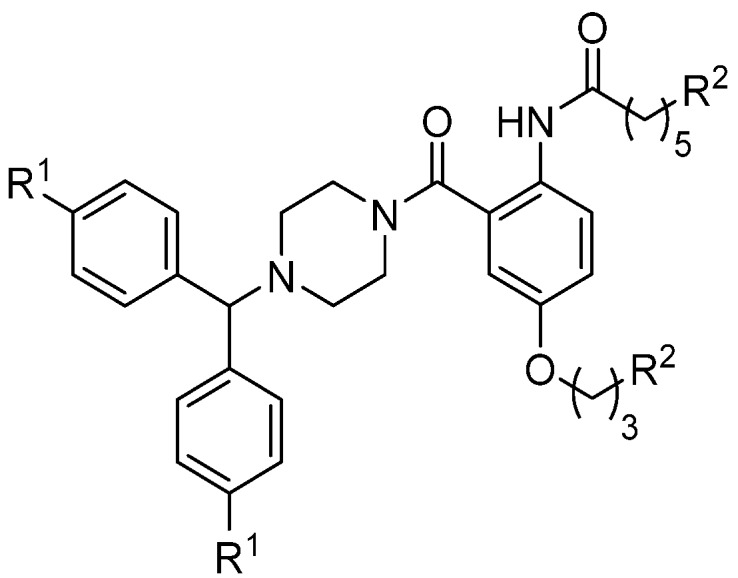
Further exploration of the anthranilamide class of compounds where the phenoxyaniline substituent has been replaced with a diphenylmethylpiperazine moiety [51].

Recognizing that effective voltage-gated calcium channel blockers Z160, a reformulated form of NP118809 [66], and Abbott’s A-1048400 [67] contain a diphenylmethylpiperazine moiety, Duggan and Tuck investigated substitution of the phenoxyaniline substituent with diphenylmethylpiperazine in the anthranilamide class of compounds [51]. Such a replacement was expected to limit the toxicity of these compounds. A number of analogues were synthesized and tested for their ability to block neuronal calcium channels in non-functional and functional assays (Figure 11). This was the first report that compared the Ca_v_2.2 activity determined using the radioligand displacement assay, the FLIPR assay (fluorescence-based calcium response assay with SH-SY5Y neuroblastoma cells) and a whole-cell patch clamp electrophysiology assay. Pleasingly, the mimetics identified to be active in the radioligand displacement assay were also found to elicit functional inhibition of intracellular calcium responses in SH-SY5Y neuroblastoma cells and calcium currents in HEK293 cells stably expressing human Ca_v_2.2 channels. These results justify the overall approach employed here to identify selective Ca_v_2.2 channel blockers—Which used the structure of ω-conotoxin GVIA to design inhibitors and the ^125^I-GVIA radioligand displacement assay as an initial screening tool for Ca_v_2.2 activity.

**Figure 12:**
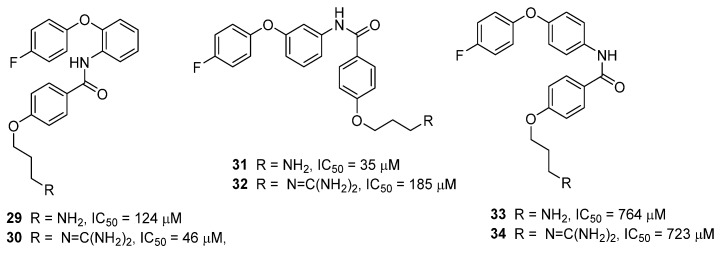
Truncation and SAR exploration of the anthranilamide scaffold. Ca_v_2.2 activity was determined using the FLIPR SH-SY5Y neuroblastoma cells assay [54].

In a recent study, Duggan and Tuck investigated molecular modifications of the anthranilamides, in the hope of improving physiochemical properties while maintaining Ca_v_2.2 activity. The anthranilamide-based mimetics were simplified by deletion of one of the amino side chain mimics and the influence of the substitution pattern of the central aromatic ring was explored [54]. Compounds **29**–**34** (Figure 12) were evaluated for their ability to inhibit Ca_v_2.2 calcium responses in SH-SY5Y cells. Compound **30** has been tested for its ability to block calcium currents in HEK293 cells stably expressing human Ca_v_2.2 channels, giving an IC_50_ of 36 µM [54]. The mode of action of this set of compounds is being further investigated in patch clamp electrophysiology experiments, and these results will be reported in the near future.

**Table:** 

Compd. No.	R^1^	R^2^	Radioligand Displacement Assay IC_50_ (μM)	SH-SY5Y Neuroblastoma Cells IC_50_ (μM)	HEK293 hCa_v_2.2 + β3 + α2δ1 IC_50_ (μM)
**25**	H	NH_2_	214	160	232
**26**	H	N=C(NH_2_)_2_	12	206	299
**27**	F	NH_2_	65	286	288
**28**	F	N=C(NH_2_)_2_	16	156	156

### 3.4. μ-Conotoxin Mimetics

**Figure 13:**
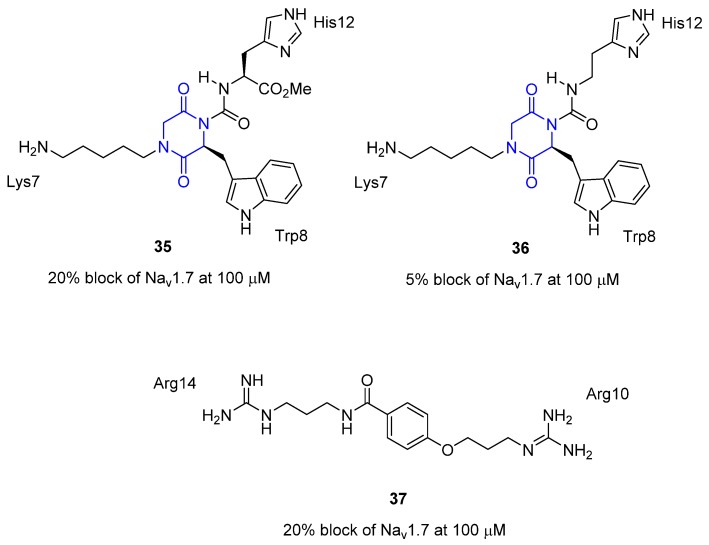
Non-peptide mimetics of μ-conotoxin KIIIA by Baell and coworkers. The diketopiperazine carboxamide scaffold is highlighted in blue in **35** and **36**. Activity is block of the Na_v_1.7 channel (patch clamp assay) [72,73].

μ-Conotoxin KIIIA, from *Conus kinoshitai*, is a **16** residue, three-disulfide bridged, peptide that blocks several voltage-gated sodium channel isoforms and has been shown to have potent analgesic activity in the mouse model for pain [68,69]. Structure–function activity studies determined that the key residues required for KIIIA’s activity against voltage-gated sodium channels were Lys7, Trp8, Arg10, Asp11, His12, and Arg14 [69]. Additionally, the solution NMR structure of μ-conotoxin KIIIA revealed that 5 of the 6 key residues (the exception was Arg14) were located on an α-helix. It was thus concluded that the α-helix plays an important role in the interaction of μ-conotoxin KIIIA with the voltage-gated sodium channels [70,71]. Recently type-III peptidomimetics of μ-conotoxin KIIIA have been designed with the aid of an *in silico de novo* strategy [72]. Using the C_α_–C_β_ bond vector approach Baell and coworkers devised a diketopiperazine carboxamide scaffold that would mimic the C_α_–C_β_ bond vectors of the residues Lys7, Trp8 and His12. A key part of the design is the use of the amide link to the His12 mimic, thus resulting in an internal H-bond that causes the molecule to adopt the conformation required to correctly mimic the C_α_–C_β_ bond vectors of the His12 sidechain. In this study two such mimetics were synthesized (compounds **35** and **36**, Figure 13) and tested for their ability to block the Na_v_1.7 channel (patch clamp assay). While both compounds only weakly blocked the Na_v_1.7 channel they are the first type-III peptidomimetics developed based on the native structure of μ-conotoxin KIIIA. It was reported that peptidomimetic **35** will serve as a template for future studies. A second scaffold, which has a relatively simple design, was developed to mimic the Arg10 and Arg14 residues in μ-conotoxin KIIIA [73]. Compound **37** blocked the Na_v_1.7 channel relatively weakly.

## 4. Snake Venom Peptides and Their Mimetics

### 4.1. Echistatin

**Figure 14:**
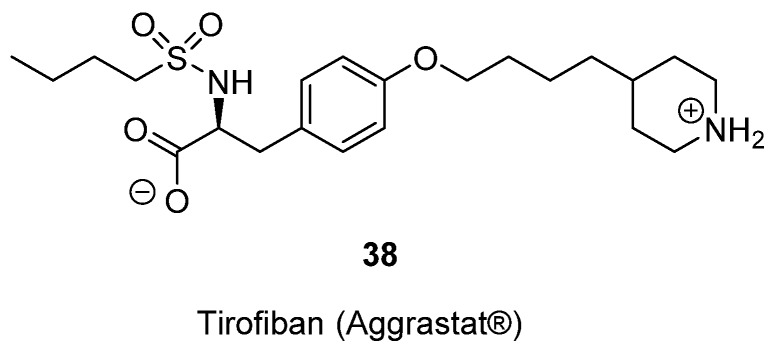
The chemical structure of Tirofiban **38**.

Tirofiban (Aggrastat^®^, MK-0383; L-700,462, **38** Figure 14) a small molecule peptidomimetic antiplatelet drug, was discovered by researchers from Merck in the early 1990s [74,75,76,77,78,79] and granted FDA approval in 1998. The development of Tirofiban was based on the 3D-structure of Echistatin (disintegrin), which is a derivative of an anticoagulant found in the venom of the African saw-scaled viper *Echis carinatus*. Researchers from Merck designed small molecule mimetics of the cell adhesion motif RGD (Arg-Gly-Asp), which was identified as the recognition element required for the antiplatelet activity possessed by Echistatin. Merck’s in-house sample collection was searched for compounds that had carboxylate and amino functionalities separated by distances of 10–20 Å. The latter corresponds to the approximate distance between the termini of the Arg and Asp side chains in RGD. Tirofiban was the end result of optimization of the initial hit from this screen [74,75].

### 4.2. Erabutoxin B

**Figure 15:**
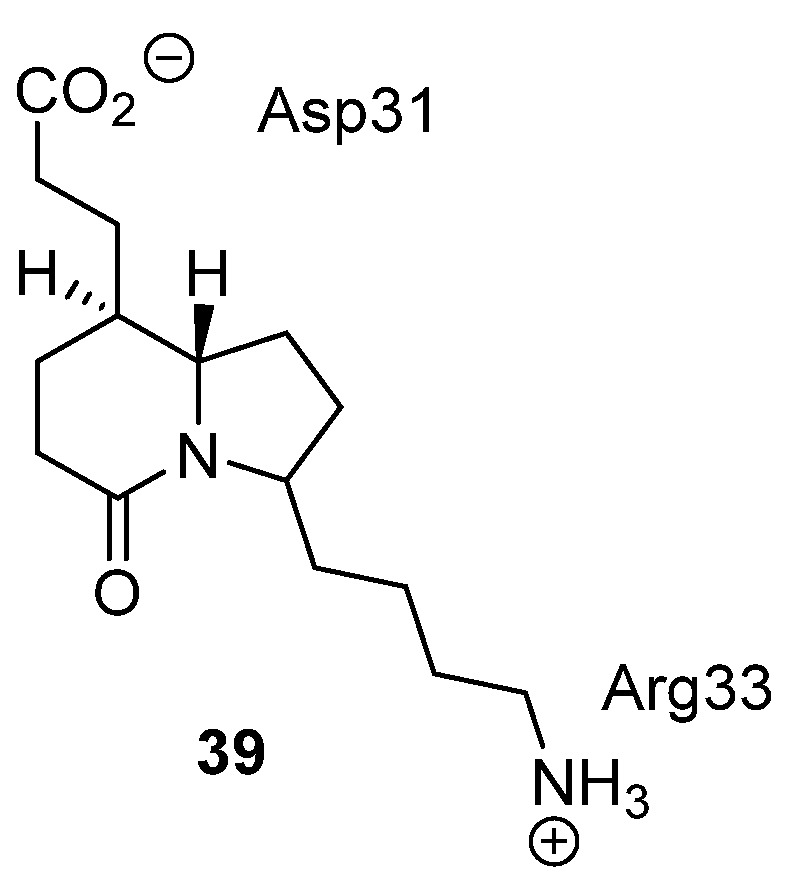
The chemical structure of an erabutoxin mimic **39**.

Erabutoxin B, found in the venom of the broad-banded blue sea snake, is a 62 amino acid protein bearing four disulfide bridges. It has high affinity for the post-synaptic nicotinic acetylcholine receptor (nAChR) [80,81]. Compounds that act on the nAChR have the potential to be treatments for nicotine addiction, to relieve the effects of Alzheimer’s disease or be muscle relaxants. Kahn and coworkers utilized the X-ray structure of erabutoxin B [82,83], and the knowledge that residues **31**–**34** (Asp, Phe, Arg, Gly) that form a β-turn are essential for binding to the nAChR, to design a type-III erabutoxin mimetic [84], guided by molecular modeling. This led to the hexahydro-indolizinone scaffold being chosen as the mimetic core. The side chain residues of Asp31 and Arg33 in erabutoxin B were appropriately mimicked as either a carboxylate or an ammonium salt respectively (compound **39**, Figure 15). This seminal work is the first report of molecular modeling guided design of a small molecule mimic of a peptide or protein found in the venom of snakes or cone snails. Unfortunately, no biological evaluation of this mimetic has been reported and hence it is unclear if this is a true type-III mimetic of erabutoxin.

## 5. Sun Anemone Toxin

**Figure 16:**
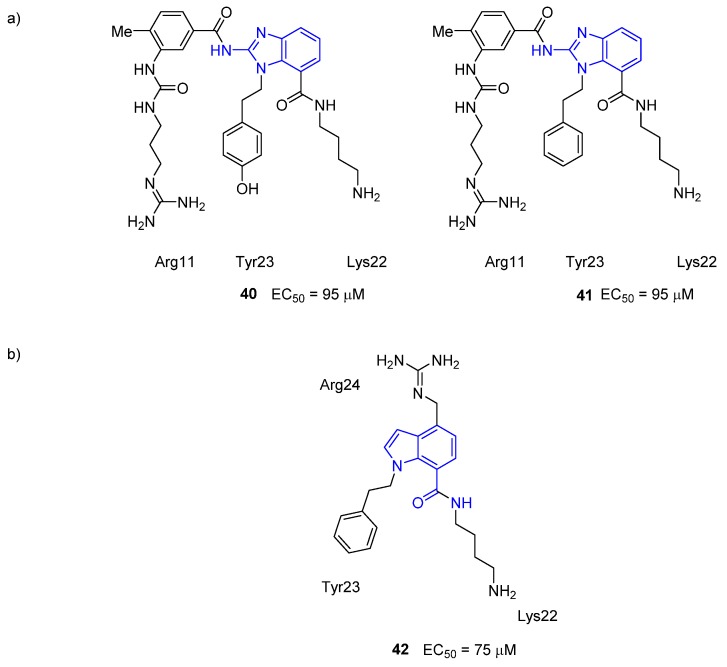
Peptidomimetics of ShK that contain the (**a**) 2-aminobenzimidazole scaffold, compounds **40** and **41** [89]; and (**b**) the *N*-alkylated indole-7-carboxamido scaffold, compound **42** [90]. The scaffold structure is highlighted in blue. Activity was determined using a voltage-gated K_v_1.3 potassium channels patch clamp assay.

Stichodactyla toxin (ShK), from the sea anemone *Stichodactyla helianthus*, is a **35** residue peptide that binds strongly to a number of voltage gated potassium channels [85]. The peptide has three disulfide linkages that produce a structurally well-defined molecule. ShK is known to have three separate binding sites [86,87]. The determination of the NMR solution structure allowed mapping of the potassium channel binding surface and thus identified the residues responsible for binding [88]. The toxin was found to contain a discontinuous (Arg11 and Phe27) and contiguous (Lys22, Tyr23 and Arg24) binding surface but its rigid nature makes it an attractive target for the rational design of type-III mimetics. Baell *et al.* utilized the previously mentioned the α,β-bond vector approach to devise suitable mimetics. Initially, the contiguous residues (Lys22, Tyr23 and Arg24) were investigated with a core *N*-benzylhydantoin scaffold. For synthetic simplicity, the Tyr sidechain mimic was replaced with a Phe mimic, as it was known that the OH group of Tyr23 is not required for binding [86]. This initial compound was inactive (structure not shown), presumably because the hydantoin ring orientates the phenyl ring (Tyr23 mimic) in an orientation that it is perpendicular to the way Tyr23 is displayed in the ShK toxin [89]. Subsequently the Lys22-Tyr23-Arg11 pharmacophore was investigated using a quinoxaline-dione ring, however this compound was also inactive [89]. Use of the 1,7-disubstituted 2-aminobenzimidazole core with appropriate functionalization allowed the development of hydroxylated and unhydroxylated compounds **40** and **41** (Figure 16). These compounds showed the same activity in voltage-gated K_v_1.3 potassium channels patch clamp assay, thus confirming that the OH group of Tyr23 is not required for binding. The biological results from the first generation of compounds enabled pharmacophore-based database mining. Three nonpeptide hits were subsequently tested for activity, but were found to be inactive. While these first generation molecules were relatively weak binders, Baell and coworkers “built on” these results with their second-generation peptidomimetics [90]. For this study, they focused on the contiguous residues (Lys22, Tyr23 and Arg24), thus allowing for a smaller mimetic. Again, using the α,β-bond vectors of the Lys22 and Tyr23 side chains from the NMR solution structure of ShK, an *N*-alkylated indole-7-carboxamido scaffold was designed (compound **42**). Further derivatization at the 4-position allowed for additional mimicry of the Arg24 sidechain residue. The activity of this compound was marginally better than that observed for the first-generation mimetics. It was noted that the production of a library of mimetics based around this the indole might lead to compounds with improved activity.

## 6. Summary and Conclusions

The stupendous number of pharmacologically active substances present in animal venoms is a largely untapped source of new bioactives. Peptides form a significant component of these actives but due to problems associated with their bioavailability, these compounds have rarely reached the clinic. The development of lower molecular weight organic compounds that mimic the action of some of these peptides—Peptidomimetics—has been one approach aimed at overcoming bioavailablity problems. Peptides found in the venom from the *Conus* marine snails have received by far the most attention in this regard. This has largely focused on the mimicry of the neuronal calcium ion channel blocking ability of the ω-conotoxins. The ω-conotoxins are exquisitely selective and potent modulators of voltage gated calcium ion channels and the design and construction of small molecules that approach the activity of these peptides has been a challenging task. Nonetheless, progress has been made with ω-conotoxin GVIA mimics, with the most recently developed compounds showing considerable promise. Mimicry of active peptides from snake and anemone venom has also been undertaken. The initial hit that led to the antiplatelet drug Tirofiban was inspired by an active peptide found in the venom of the broad-banded blue sea snake.

There are numerous avenues to the discovery of hits with the potential to be developed into potent bioactives, including the screening of natural and synthetic compound collections, analoging of known drugs and fragment-based approaches. Where little is known about the structure of the biological target, which is the case with many neuronal ion channels, the mimicry of venom peptides remains an attractive option that is expected to be fruitful for many years to come.
